# Auxin-Induced Adventitious Root Formation in Nodal Cuttings of *Camellia sinensis*

**DOI:** 10.3390/ijms20194817

**Published:** 2019-09-27

**Authors:** Kang Wei, Li Ruan, Liyuan Wang, Hao Cheng

**Affiliations:** Key Laboratory of Tea Biology and Resources Utilization, Ministry of Agriculture, National Center for Tea Improvement, Tea Research Institute Chinese Academy of Agricultural Sciences (TRICAAS), Hangzhou 310008, China; weikang@tricaas.com (K.W.); ruanli@tricaas.com (L.R.)

**Keywords:** adventitious root formation, auxins, tea cuttings, nitric oxide, hydrogen peroxide, cell wall

## Abstract

Adventitious root (AR) formation is essential for the successful propagation of *Camellia sinensis* and auxins play promotive effects on this process. Nowadays, the mechanism of auxin-induced AR formation in tea cuttings is widely studied. However, a lack of global view of the underlying mechanism has largely inhibited further studies. In this paper, recent advances including endogenous hormone changes, nitric oxide (NO) and hydrogen peroxide (H_2_O_2_) signals, secondary metabolism, cell wall reconstruction, and mechanisms involved in auxin signaling are reviewed. A further time course analysis of transcriptome changes in tea cuttings during AR formation is also suggested to deepen our understanding. The purpose of this paper is to offer an overview on the most recent developments especially on those key aspects affected by auxins and that play important roles in AR formation in tea plants.

## 1. Introduction

Tea, the product of *Camellia sinensis* (L.) O. Kuntze. (Family: Theaceae), is one of the most popular and consumed beverages worldwide [1]. It possesses abundant secondary metabolites like polyphenols, theanine, and caffeine, which have many beneficial effects to human health, such as prevention of cancer [2], obesity [3], and cardiovascular diseases [4]. Moreover, some special tea cultivars with new beneficial effects, such as purple tea [1,5], albino tea [6], and high gallotannin tea cultivars [7] have been bred in recent years. The popularity of these teas facilitates their spread in many countries.

Vegetative propagation is widely used in forestry. Among vegetative propagation methods, including budding, grafting, layering, and cutting, the method using nodal cuttings from good genotypes is the most efficient and economical way to produce large numbers of homogeneous plants [8]. Usually a stem or a leaf needs to be able to survive independently from the mother plant, and the regeneration and development of new roots are essentially important. These roots, which grow from organs other than the root system after the embryonic stage, are called adventitious roots. Up to date, tea propagation is mainly dependent on nodal cuttings, which ensure the quality stability of new cultivars. During this process, adventitious root (AR) formation is the prerequisite for the successful propagation. AR formation is a developmental process, where new roots are generated spontaneously or in response to certain stimuli from stems, leaves, or non-pericycle tissues of older roots [9]. Unlike lateral roots, ARs can develop either from pericycle cells or from various cell types and tissues, which depend on the plant species and environmental stimuli involved [10]. For example, the ARs of *Arabidopsis* may initiate from xylem pole pericycle cells in both intact and de-rooted hypocotyls, while the ARs of rice may initiate from the peripheral vascular cylinder. The formation of ARs is a strictly controlled developmental process, which generally includes the following three steps: (1) Cell specification and reprogramming, in which differentiated cells acquire new cell fate programs leading to the specification of AR founder cells; (2) initiation, in which the AR founder cells undergo successive cell divisions leading to the formation of primordia; and (3) primordia emergence and outgrowth. Slow or insufficient rooting leads to the death of whole plants [11]. Thereby, many root stimulators were developed to overcome this problem. In order to overcome the difficulties caused by the loss or weakening of AR development function, and to better understand the whole process of AR development, many physiological, biochemical, and molecular studies have been carried out on model plants and cash crops. These studies indicate that AR formation is a heritable quantitative genetic trait, which is controlled by multiple endogenous and environmental factors. Among these factors, auxin, light, temperature, and nutrient elements are the most important. The commercialization of tea cuttings is restricted by the serious loss of the rooting process [6]. Phytohormones, together with many other internal and external stimuli, coordinate and guide every step of AR formation, from the first event of cell reprogramming until emergence and outgrowth. Obviously, the core factor regulating the initial growth of AR is the interaction of phytohormones. Among these phytohormones, auxins play a leading role [11]. Auxins, which include 2,4-dichlorophenoxy-acetic acid (2,4-D), indole-3-butyric acid (IBA), indole-3-acetic acid (IAA), and α-naphthalene acetic acid (NAA), are the most widely used root stimulators in practice. Auxins act with an array of other phytohormones through very complex crosstalk, modulating each other’s levels and actions at every level: Biosynthesis, metabolism, transport, and signaling [12]. The recent studies showed that auxins greatly promote rooting efficiency of tea cuttings [13,14,15,16]. In the past two decades, extensive studies were carried out to explore the underlying mechanism. Although our knowledge about this process is still fragmentary, some key points of the basic mechanism are becoming clear. This review focuses on these key aspects affected by auxins and discusses their importance in AR formation in tea plants.

## 2. Endogenous Hormone Changes

Recent studies in different plants suggest that complex interactions of many hormones occur during AR formation, and auxin is likely to take part in almost all of the interactions. In addition, the interaction of different hormones regulates the successive stages of AR development, including the induction, initiation, and expression of AR formation [17]. Such interactions are difficult to study because they vary depending on the rooting conditions, the species, or whether the entire plant, stem cuttings, or other alternative systems are performed.

External application of auxins firstly affects the balance of endogenous hormones, which leads to changes in the plant developmental process [18,19]. It has long been established that different kinds of auxins play different roles in promoting the AR formation of different species [11,20]. IAA, NAA, and IBA have a promoting effect on the AR formation of the tea tree cuttings. Different phytohormone concentrations have different effects on AR formation. Among the above three phytohormones, IBA is superior to the other two phytohormones [15]. As IBA is the most effective and stable promoter of AR formation, it is widely used in clonal propagation. It is generally believed that IBA promotes AR formation mainly through the conversion of IBA to IAA [12,21]. In tea plants, RNA-Seq analysis of cuttings treated with or without IBA showed that genes involved in auxin homeostasis were seriously affected [15]. Furthermore, most of these genes functioned as suppressors of endogenous IAA. For example, the expressions of two indole-3-acetic acid-amido synthetase (GH3) genes and an indole-3-acetate O-methyltransferase gene were 157-, 92-, and 30-fold higher in IBA-treated tea cuttings. GH3 is involved in the conjugation of free auxin with amino acids and overexpression of GH3 results in auxin-deficiency in plants [22,23]. Meanwhile, indole-3-acetate O-methyltransferase, also named as IAA carboxyl methyltransferase is able to methylate the free carboxyl group of IAA to produce an inactive hormone [24]. Our recent study also showed that external application of NAA significantly decreased the endogenous IAA level in tea cuttings. These results reveal that an antagonistic mechanism in tea cuttings is induced by external application of auxins. IBA or NAA generally show better promotive effects on the expressions of key genes involved in AR formation than IAA in tea plants [14]. Based on these studies, it can be concluded that: (1) Auxins are important for AR formation in tea cuttings; (2) IBA or NAA might be able to directly affect the expressions of key genes involved in AR formation rather than via conversion to IAA; and (3) the antagonistic mechanism reduces the endogenous IAA level, but might not affect IBA or NAA levels. As in agricultural practices, the promotive effect of IAA on AR formation is generally less than that of IBA or NAA, it might be correlated with the antagonistic mechanism.

Cytokinin is another important hormone affected by external auxin application and associated with AR formation in tea cuttings. The low ratio of auxin vs. cytokinin levels will lead to weak rooting [25]. External application of NAA also significantly decreases trans-zeatin riboside (the most active cytokinin) content in the basal parts of cuttings according to our recent study. Cytokinin is considered as a type of inhibitor for auxins which largely suppresses the AR formation in many plant species [8]. RNA-Seq analysis also showed that exogenous IBA inhibited cytokinin biosynthetic genes but induced degradation genes in tea cuttings [15]. For example, an adenylate isopentenyltransferase gene and two cytokinin hydroxylase genes were inhibited but two cytokinin oxidase genes were induced by IBA. In model plants, cytokinin–auxin crosstalk had already been proved to affect plant organ differentiation [26]. A high ratio of in vivo cytokinin to auxin level promotes shoot formation, but low ratio of in vivo cytokinin to auxin facilitates root formation. Therefore, the decrease of cytokinin in auxin-treated tea cuttings agrees with findings in model plants and should also be a key factor of AR formation.

Ethylene (ET) was shown to positively regulate AR formation, which was probably through the modulation of auxin transport. It has been reported that ET can promote AR development in cuttings of different plant species [27]. Moreover, this promoting effect is especially reflected in the interaction between ET and auxin, although the corresponding mechanisms are unclear yet [28]. Recently, it has been proposed that there are complex interactions between ET and auxin in the regulation of AR formation [29]. Two genes *ANTHRANILATE SYNTHASE ALPHA 1/WEAK ETHYLENE INSENSITIVE 2 (ASA1/WEI2)* and *ANTHRANILATE SYNTHASE BETA 1/WEAK ETHYLENE INSENSITIVE 7 (ASB1/WEI7*) involved in auxin synthesis also take part in the regulation of ET signal [29]. ET may affect AR formation by changing the perception of auxin, as the suppressor mutated in the *RUB-CONJUGATING ENZYME1 (RCE1)* gene still retains the high IAA content of *superroot2-1 (sur2-1)* [11]. The isolation of these mutants as suppressors of *sur2* indicates that the cross regulation of auxin and ET take part in the AR formation process. The AR formation regulated by the interactions between auxin and ET in tea plants remains unreported. Whether ET is important for AR formation in tea plants still remains unclear. Therefore, further research in this field is crucial for tea scientists.

Brassinosteroid (BR) signaling might also play a key role in AR formation. Both auxins and BRs are interdependent and show overlapped functions in cell expansion and proliferation in *Arabidopsis* [30]. Furthermore, BRs were reported to be closely correlated with auxin growth responses like gravitropic response and lateral root formation [31,32]. In tomato, the primary roots of *bushy* mutants were much shorter than their counterparts in the “Micro-Tom” background, which were correlated with the internal BR contents [33]. In tea cuttings, IBA treatment strongly induced four bHLH135 genes, which were potentially involved in BR signaling [15,34]. However, as the BR content in tea cuttings had not been determined yet, more research about the real function of BR in tea cuttings needs to be done. Other genes involved in the abscisic acid (ABA) and gibberellin (GA) pathways were also found to be affected by IBA [15]. However, our recent experiment showed that the endogenous ABA, GA1, and GA3 were little affected in tea cuttings. Therefore, they were not considered to play key roles in AR formation.

## 3. NO and H_2_O_2_ Signals

Both nitric oxide (NO) and hydrogen peroxide (H_2_O_2_) are considered as key signals involved in auxin-induced AR formation in plant species [9,35]. In most cases, they occur in parallel and show strong interactions [36,37]. For herbaceous plants, exogenous auxin application causes the bursts of NO and H_2_O_2_ [9,38]. Moreover, exogenous sodium nitroprusside (NO-donor) and H_2_O_2_ are able to mimic the effect of auxins in AR formation in herbaceous plants [9,39]. However, few studies have been performed on the roles of NO and H_2_O_2_ in AR formation in woody plants. Therefore, we used the fluorescent probe diaminofluorescein diacetate (DAF-2DA) and 2′,7′-dichlorodihydrofluorescein diacetate (DCF-DA) to in situ detect NO and H_2_O_2_ in IBA-induced AR formation in tea cuttings [16]. Both NO and H_2_O_2_ signals were not directly induced by exogenous IBA application, as they were not detected before the initiation phase of tea cuttings. The bursts of NO and H_2_O_2_ were only observed in the rooting parts of tea cuttings irrespective of IBA treatment. Furthermore, our previous experiment found exogenous sodium nitroprusside (NO-donor) and H_2_O_2_ did not show any promotive effect on AR formation, but lead to plant death in tea cuttings. These results illustrate that NO and H_2_O_2_ participate in AR formation in tea cuttings, but their roles might not be that important as compared with those in herbaceous plants. Both signals play important roles in the initial and expression phases, but not the induction phase for tea cuttings [16]. Therefore, the factor of the rooting phase should be taken into consideration in tea cuttings. Moreover, we hypothesized that the promotive effect of exogenous NO and H_2_O_2_ on AR formation of herbaceous plants might be correlated with their short induction phase [16]. In terms of tea plants, the induction phase is much longer and NO and H_2_O_2_ might not be involved in that preparation stage. Therefore, that might explain why exogenous application of them did not show any obvious effects on AR formation.

## 4. Secondary Metabolism

Secondary metabolism, especially phenolic compounds, have long been considered to be linked to auxins and involved in AR formation [10]. In stem slices cut from apple micro-shoots, it was found that di- and polyphenolic compounds were induced by auxins and promoted AR formation [40]. In tea plants, the total phenolic content was also induced by IBA in cuttings [13]. Because phenolics may play a key role in inducing the development of AR, auxin inhibits or promotes the root development of tea cuttings by changing the content of phenolic compounds (as regulators of peroxidase and polyphenolic oxidase activity). Wei et al. (2013) also reported the key flavonoid biosynthetic genes were induced by IBA in tea cuttings [14]. These results confirmed the potential importance of flavonoids in AR formation of tea cuttings. The effects of flavonoids on AR formation might be correlated with their functions in auxin transport, as many flavonoids interacted with auxin carriers [41]. However, which flavonoid components are induced by auxins in tea cuttings? What are their roles in AR formation? The answers to these questions are still unavailable. Therefore, relative metabolite profiling studies should be performed in the future, which will deepen our understanding in this field.

Besides flavonoids, isoprenoid biosynthesis was also found to be affected by IBA in tea cuttings [15]. The 2-C-methyl-d-erythritol 4-phosphate (MEP) pathway-associated genes were inhibited, while a key gene (*HMG1*) involved in the mevalonate (MVA) pathway was induced by IBA [15]. The MEP and MVA pathways are distinct, compartmentalized pathways present in cytoplasm and plastids, respectively [42]. The MEP pathway is involved in the biosynthesis of terpenes, carotenoids, and the phytol group of chlorophylls [43]. While the MVA pathway is responsible for the production of sterols, sesquiterpenes, and the side chain of ubiquinone [44]. The *HMG1* gene in the MEP pathway is closely associated with pigment formation, and plants treated with inhibitors of MEP pathway exhibit albino phenotypes [45]. On the other hand, the MVA pathway is correlated with plant development [46]. The gene expression profiles associated with the MVA and MEP pathways under IBA treatment might play important roles in the inhibition of shoot development and facilitation of AR formation in tea cuttings, which is also worth further research.

## 5. Cell Wall Reconstruction

Cell walls acting as exoskeleton structures surrounding certain cells undergo lots of changes during AR formation. The degradation, loosening, and stretching of cell walls are essential for cell elongation and division, which are the prerequisite for AR initiation [47,48]. Cell walls consist of cellulose microfibrils, hemicelluloses, pectins, lignin, and structural proteins [49].

Cellulose microfibrils are the largest cell wall polysaccharides. They are much more ordered than any other component of the primary cell wall, which provide the stiffness and load-bearing properties [50]. Hemicelluloses and pectins are the most abundant components in cell walls. Hemicelluloses affect cell wall extensibility and stiffness, thereby are considered to participate in cell wall extension during cell elongation [51]. On the other hand, recent studies showed that pectins regulate wall porosity and hydration, which leads to wall swelling and affects wall thickness [48]. Furthermore, pectins can also influence the alignment of cellulose microfibrils and form the middle lamella, an adhesive compartment between cell walls, which affects wall extensibility [52]. Lignin is a class of complex organic polymers which are particularly important in the formation of cell walls in woody species, as they offer rigidity and do not rot easily.

Moreover, the cell wall contains various structural proteins, which control its growth and expansion. For example, EXTENSINs are well-characterized proteins involved in controlling wall expansion. They are able to disrupt hydrogen bonds between cellulose microfibrils and facilitate cell wall loosening [53]. Cell wall weakening genes such as cell wall-associated hydrolase and protein *WALLS ARE THIN 1 (WAT1)* genes also perform similar functions. For example, the expression of WAT1 is preferentially associated with vascular tissues, including developing xylem vessels and fibers. The knock-out of WAT1 results in a defect of cell elongation [54]. During AR formation, auxins have essential functions in apoplast acidification and activation of key wall-loosening proteins [55]. Our previous study showed that 40 genes associated with cell wall organization were highly affected by IBA in tea cuttings, which included eight expansins, 15 cell wall-associated genes, and several cell wall-weakening genes, like *WAT1* [15]. The activation of these genes might act as the key factors affecting rooting abilities.

Furthermore, for hardwood cuttings, the degree of lignification is negatively correlated with the rooting ability [56]. A similar situation was identified in tea propagation according to agricultural practices. The promotive effect of auxins on AR formation was also worse in tea cuttings with a high degree of lignification. Therefore, we hypothesized that cell wall reconstruction as well as loosening of xylem vessels and fibers are not only the early step of AR initiation in tea cuttings, but also the limiting factors for rooting abilities in tea cuttings.

## 6. The Modulation of Auxin Homeostasis and Signaling

In model plants, some genes, such as *SUR1* and *SUR2*, lead to the overproduction of auxin [57]. The rooting abilities of plants depend on their ability to bind auxin to their corresponding inactive forms. In the cellular responses, the auxin signals cause different auxin concentrations. In the recent years, the molecular mechanisms of auxin signal regulation during AR development has been gradually deepened. For example, the arrest of auxin signal causes the failure of root emergence in rice [58]. A balance of the three different *AUXIN RESPONSE FACTORs (ARFs;* i.e., *AtARF6, AtARF8,* and *AtARF17*) controls the AR formation [59]. In addition, auxin signals are correlated with light signals. After transferring to the light, the expressions of *ARF6* and *ARF8* increase in the vascular tissue of the hypocotyl, whereas *ARF17* expression decreases [60]. In tea plants, relevant researches are still in the initial stage; in particular, whether these genes are the key factors involved in difficult rooting tea cultivars remains elusive. In the future, time course-dependent changes in the transcriptome of AR formation in tea cuttings should be carried out, which would offer a global view of the mechanism regulating AR formation in tea plants.

## 7. Key Genes to Be Identified in Auxin-Mediated AR Formation

As a key model plant, recent studies in Arabidopsis could offer us a general framework of auxin-mediated AR formation, which will also shed new light on future studies of AR formation in tea cuttings. In *A. thaliana*, AR initiation is also controlled by auxin, which includes four main cell fate transition steps [61,62]. The first step involves the activation of *WUSCHEL-RELATED HOMEOBOX11* and *12* (*WOX11/12*) by auxin and results in the formation of root founder cells from regeneration-competent cells [61,63,64]. The second step is the activations of *WOX5/7* and *LATERAL ORGAN BOUNDARIES DOMAIN16* (*LBD16*) by *WOX11/12* and leads to the formation of root primordium cells from root founder cells [61,65,66]. The third step is to pattern a root apical meristem via continuous cell division. In this step, the expression of *LBD16* is gradually decreased in the meristem and many meristematic genes, like *SHOOT ROOT* (*SHR*) and *SCARECROW* (*SCR*) participate in this process [67]. The fourth step is the formation of mature AR tip and a *NAC1* (petunia NAM and Arabidopsis ATAF1, ATAF2, and CUC2) transcription factor might be involved in modulating the cellular environment to affect the emergence of AR tips [62,68]. The identification of these genes provided key references for further studies in woody plants, especially tea plant. More attentions to their homozygous genes in tea plant will facilitate our understanding of their potential roles in AR formation of tea cuttings.

## 8. Conclusions

**Figure 1:**
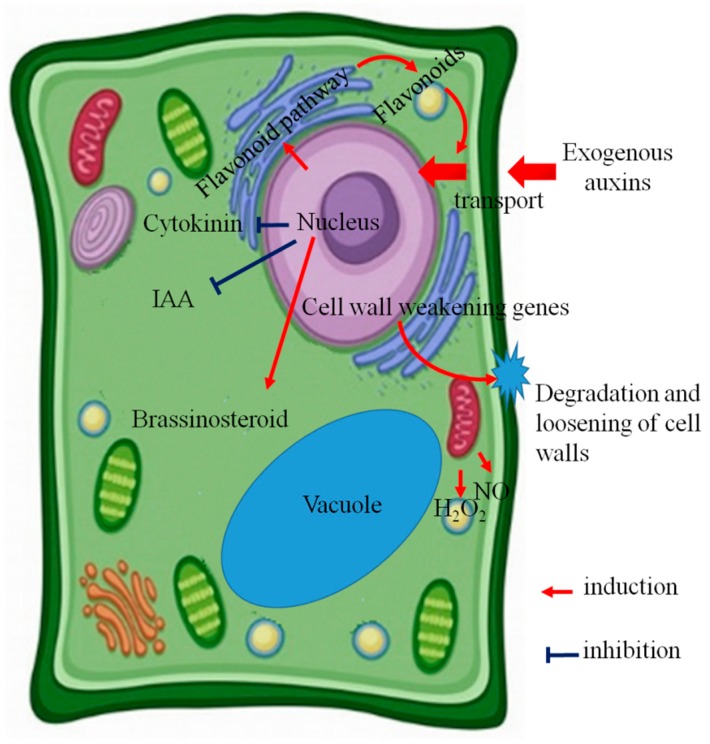
The potential mechanisms involved in auxin-induced adventitious root formation in tea cuttings. H_2_O_2_, hydrogen peroxide; IAA, indole-3-acetic acid; NO, nitric oxide.

In summary, auxins significantly improve the AR initiation of tea cuttings. The potential mechanisms involved in auxin-induced AR formation in tea cuttings were shown in Figure 1. Exogenous auxins are transported into the nucleus. At elevated nuclear auxin levels, transcriptional programs are triggered (Figure 1). Many genes involved in internal hormone balance are affected, which result in the decrease of endogenous IAA and cytokinin, but the increase of brassinosteroid. Auxins facilitate cell wall degradation, loosening, and stretching, which might be the limiting factors for rooting abilities. A secondary mechanism induced by auxins has a promotive effect on AR formation, which is worth further research. NO and H_2_O_2_ signals were only detected in the initial and expression phases, but not the induction phase, indicating that they only play important roles in the later stages of AR formation in tea cuttings. The roles of other pathways, like polar transport of auxin and auxin signaling in AR formation, and key genes involved in cell fate transitions still remain elusive. Some directions for future study were also suggested, which would facilitate our understanding of AR formation in tea cuttings.

## Abbreviations

AR	Adventitious root
NO	Nitric oxide
H_2_O_2_	Hydrogen peroxide
2,4-D	2,4-dichlorophenoxy-acetic acid
IBA	Indole-3-butyric acid
IAA	Indole-3-acetic acid
NAA	α-naphthalene acetic acid
BR	Brassinosteroid
ABA	Abscisic acid
SURRCE1	Super root rub-conjugating enzyme1
ARF	Auxin response factor
GA	Gibberellin
ET	Ethylene
NPA	Naphthylphthalamic acid
DAF-2DA	Diaminofluorescein diacetate
DCF-DA	2′,7′-dichlorodihydrofluorescein diacetate
